# A Scoping Review of Citizen Science Approaches in Chronic Disease Prevention

**DOI:** 10.3389/fpubh.2022.743348

**Published:** 2022-05-09

**Authors:** Leah Marks, Yvonne Laird, Helen Trevena, Ben J. Smith, Samantha Rowbotham

**Affiliations:** ^1^Menzies Centre for Health Policy and Economics, School of Public Health, Faculty of Medicine and Health, The University of Sydney, Sydney, NSW, Australia; ^2^The Australian Prevention Partnership Centre, The Sax Institute, Sydney, NSW, Australia; ^3^Charles Perkins Centre, The University of Sydney, Sydney, NSW, Australia; ^4^Prevention Research Collaboration, School of Public Health, Faculty of Medicine and Health, The University of Sydney, Sydney, NSW, Australia

**Keywords:** citizen science (CS), community engagement (CE), participatory research (PR), public health, chronic disease prevention, health promotion, health policy

## Abstract

**Background:**

Citizen science approaches, which involve members of the public as active collaborators in scientific research, are increasingly being recognized for their potential benefits in chronic disease prevention. However, understanding the potential applicability, feasibility and impacts of these approaches is necessary if they are to be more widely used. This study aimed to synthesize research that has applied and evaluated citizen science approaches in chronic disease prevention and identify key questions, gaps, and opportunities to inform future work in this field.

**Methods:**

We searched six databases (Scopus, Medline, Embase, PsycInfo, PubMed, and CINAHL) in January 2022 to identify articles on the use of citizen science in prevention. We extracted and synthesized data on key characteristics of citizen science projects, including topics, aims and level of involvement of citizen scientists, as well as methods and findings of evaluations of these projects.

**Results:**

Eighty-one articles reported on citizen science across a variety of health issues, predominantly physical activity and/or nutrition. Projects primarily aimed to identify problems from the perspective of community members; generate and prioritize solutions; develop, test or evaluate interventions; or build community capacity. Most projects were small-scale, and few were co-produced with policy or practice stakeholders. While around half of projects included an evaluation component, overall, there was a lack of robust, in-depth evaluations of the processes and impacts of citizen science projects.

**Conclusions:**

Citizen science approaches are increasingly being used in chronic disease prevention to identify and prioritize community-focused solutions, mobilize support and advocacy, and empower communities to take action to support their health and wellbeing. However, to realize the potential of this approach more attention needs to be paid to demonstrating the feasibility of using citizen science approaches at scale, and to rigorous evaluation of impacts from using these approaches for the diverse stakeholders involved.

## Background

Preventable chronic diseases (e.g., cardiovascular diseases, cancer, diabetes, chronic respiratory diseases and mental ill-health) are the leading cause of death and disability worldwide (1). Unhealthy diet, physical inactivity, alcohol and tobacco use and air pollution are major modifiable risk factors for many chronic diseases (2), and are shaped by interactions between complex behavioral, environmental and cultural conditions in which population groups live, work, play and age (3). Effective prevention of chronic diseases (hereafter “prevention”) requires sustained and coordinated actions delivered at multiple levels to create healthier environments and reduce preventable risks of chronic disease. Yet, feasible, acceptable, and sustainable actions are often difficult to implement, and creating health-promoting environments requires the support and advocacy of diverse stakeholders, including the public. Engaging members of the public in the design, implementation and evaluation of prevention initiatives is a recognized mechanism of ensuring that health policies and programs adequately reflect the needs, concerns, and perspectives of communities (4, 5). There is growing recognition of the need for collaborative and inclusive approaches both to scientific research (6, 7), and decision making affecting public health and wellbeing (8–12).

**Figure 1:**
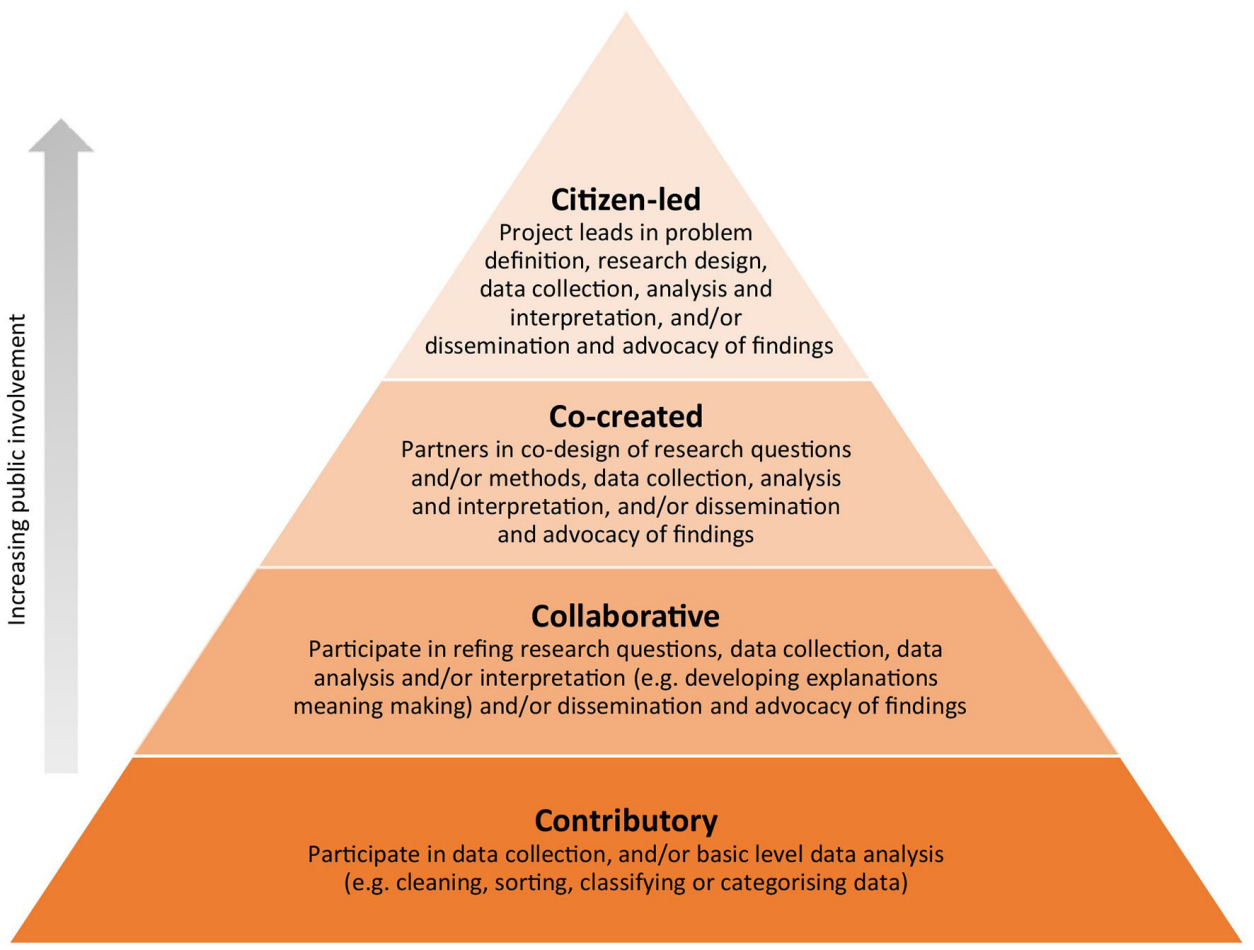
Four models of citizen science characterized by increasing levels of public involvement in the research process. Adapted from Den Broeder et al. (19) and English et al. (21).

Citizen science approaches actively involve members of the public (known as “*citizen scientists*”) as collaborators in scientific research (13, 14), for example in formulating research questions, collecting data or interpreting and acting upon findings. Citizen science is part of a broader movement toward open science, open government and participatory democracy (15), and reflects a shift from research done *for* the public to research conducted *with* or *by* them (16, 17), recognizing the voice, lived experience and expertise community members bring. Although the definition of what constitutes citizen science is evolving (15), this can encompass a broad spectrum of practices, initiatives and activities to engage the public in the research process in many ways (18). One way of classifying citizen science approaches is to use a continuum of citizen scientists' level of involvement in the research process. This ranges from “*contributory”* approaches in which citizen scientists mainly contribute to collecting and/or analyzing data, to *collaborative* approaches where citizens have opportunities to contribute to various stages of the research process in collaboration with the researchers, through to *co-created* and *citizen-led approaches*, which are characterized by higher levels of control and leadership by citizen scientists in the design, delivery and translation of projects (19, 20) (see Figure 1 for an overview of the four approaches).

**Figure 2:**
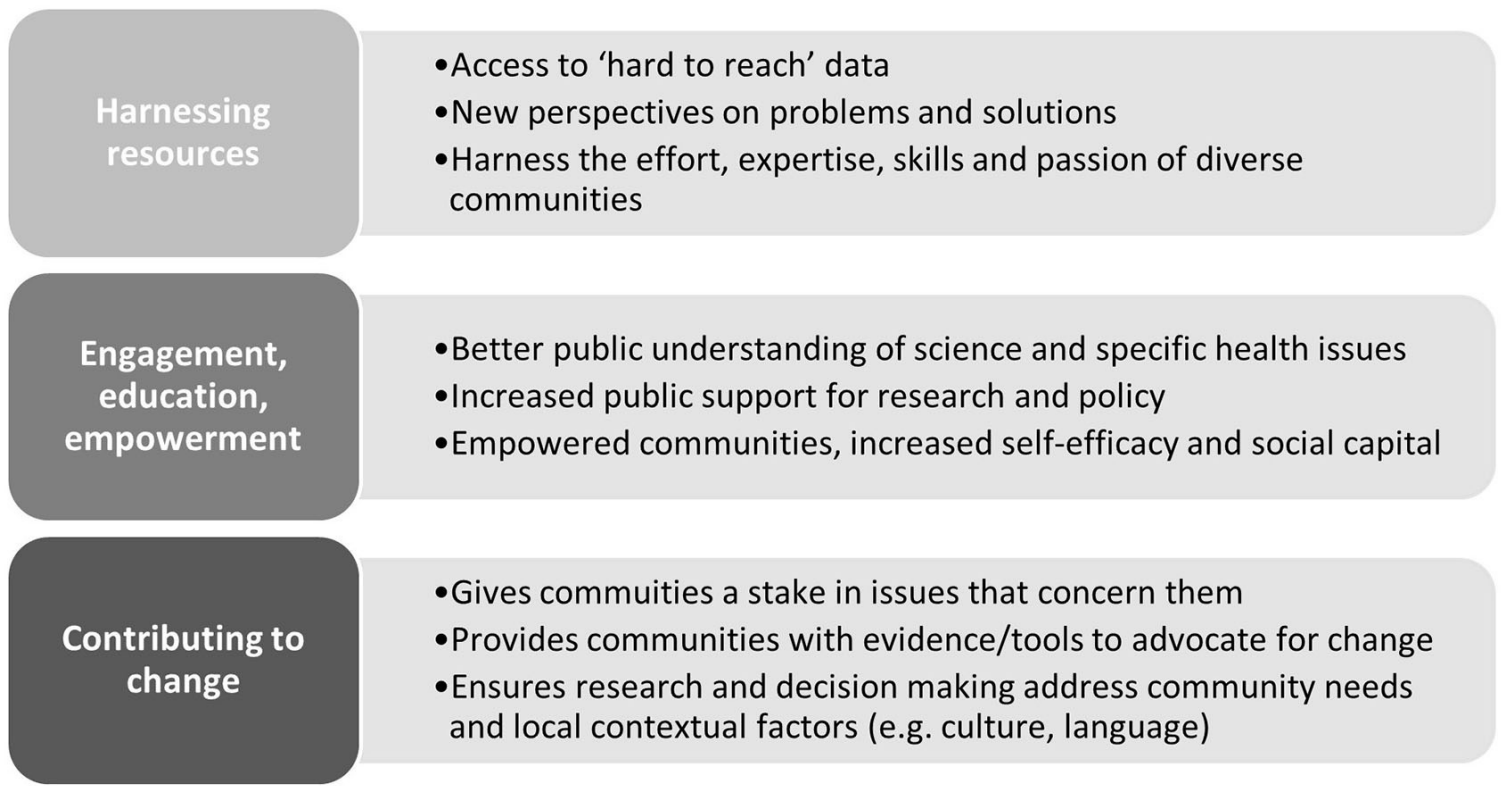
Potential applications of citizen science in chronic disease prevention (25).

While originating in the natural sciences, citizen science approaches have grown rapidly in recent years across a range of disciplines, including public health (22–24). Citizen science builds on long standing traditions of public engagement in health promotion and shares commonalities with other approaches to engaging the public in research (e.g., community-based participatory research, participatory action research, and crowdsourcing). By harnessing the effort and expertise that community members bring to the research process, citizen science may offer opportunities to strengthen prevention initiatives (see Figure 2). It is increasingly recognized as a valuable approach to generate new knowledge to address real-world problems (26) and to mobilize public support for preventive actions to bring about change at local, state and national levels. As such, citizen science is gaining traction not only as a way to generate data but as a way to better engage the public in policy processes, ensure actions are oriented toward addressing issues of community and societal relevance, and ultimately, to shape policy and practice more broadly (27–29). Importantly, citizen science has the potential to promote meaningful public engagement with prevention issues and solutions. In turn, strengthening partnerships between citizens, researchers, practitioners and policymakers to potentiate the effectiveness of strategies to promote health and wellbeing (17, 19, 30–33).

Given the growing interest in citizen science approaches amongst researchers, policy and practice stakeholders and funders in chronic disease prevention (19, 20, 31, 33–37), this review aimed to map how citizen science approaches have been applied in this field, in order to identify gaps and opportunities for future work. In particular we sought to identify the key topics and questions being addressed using citizen science and the way in which citizen science approaches are applied. We were also interested in understanding whether and how citizen science projects in prevention have been evaluated for feasibility and impacts.

The review questions were:

a) How have citizen science approaches been applied within chronic disease prevention? including in what areas of prevention, the methods used and ways in which members of the public are involved.
b) How have citizen science projects been evaluated in chronic disease prevention?

## Methods

This scoping review followed the five-stage process outlined by Arksey and O'Malley (38) (1) identifying the research question, (2) identifying relevant studies, (3) study selection, (4) charting the data, and (5) collating, summarizing, and reporting the results. The review title was registered on the Joanna Briggs database of systematic reviews and is reported in line with PRISMA-ScR reporting guidance (see Supplementary File 1 for a copy of the completed PRISMA-ScR checklist).

### Search Strategy

The search strategy was developed in consultation with a research librarian. We searched six electronic databases: Scopus ([1970–]), Medline (1946–), Embase ([1947–]), PsycInfo (1967–) PubMed and CINAHL for articles published until January 2022, on citizen science in chronic disease prevention. As we were interested in how approaches explicitly referred to as “citizen science” have been applied in prevention, we combined the term “citizen science” with subject headings and free-text terms relating to health topics such as physical activity, smoking, diet and liveability (see Supplementary File 2 for search terms).

We also hand searched the reference lists of included articles and contacted experts via personal emails, Twitter, and messages to citizen science listserv groups and relevant organizations (including UK National Co-ordinating Centre for Public Engagement and Citizen Science Associations in Australia, Europe and the USA) to request relevant publications for inclusion.

### Study Selection

In line with the recommendations of Levac et al. (39) the criteria for study inclusion were refined through discussion amongst the research team in an iterative manner. Articles were included if they met the following criteria: (a) had a focus on chronic disease prevention (including research, policies and programs to reduce chronic disease and/or associated risk factors); (b) reported on the application or evaluation of a citizen science approach; and (c) explicitly used the term “citizen science” to refer to the approach used. Articles were considered as reporting an evaluation component if they examined processes (e.g., citizen scientist's motivations and experiences, feasibility, acceptability or utility of citizen science approaches), and/or impacts of citizen science projects (e.g., for citizen scientists, for policy and practice). Only peer-reviewed research articles published in English were included. Articles were excluded based on the following criteria: (a) protocols, editorials, reviews, theses/dissertations and conference papers; (b) absence of a clear link to prevention in the study background or aims. While important for the prevention of chronic disease, projects using citizen science for monitoring air and water quality were not included within this review as literature on the use of citizen science for environmental health has been comprehensively summarized elsewhere (21).

### Article Screening

**Figure 3:**
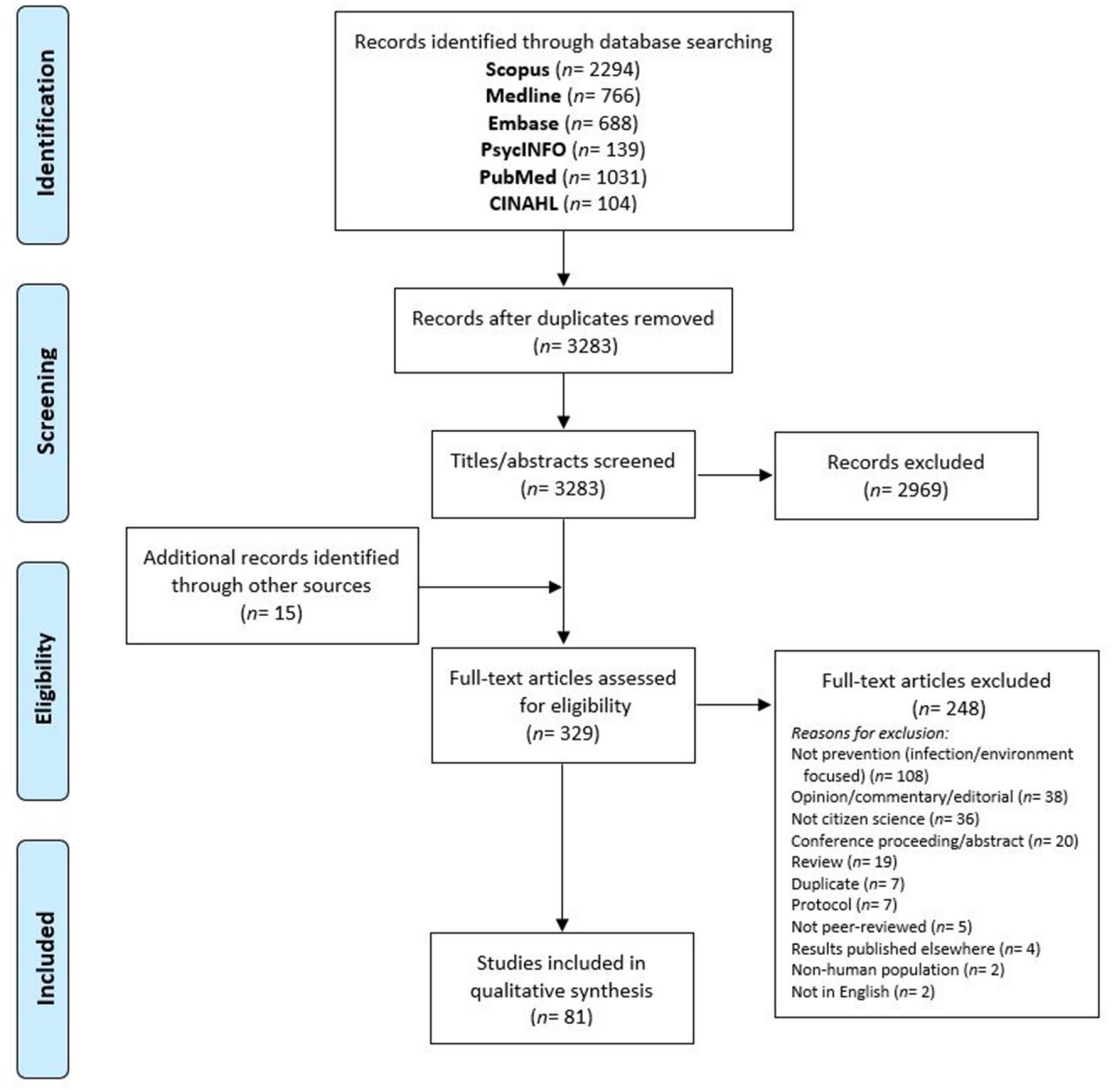
PRISMA flow diagram.

Database search records were imported into Covidence (40) for de-duplication and screening. Titles and abstracts were screened independently by two reviewers (LM, YL, and SR). Following this, full-text articles were retrieved and independently screened by two reviewers (LM, YL), with a third reviewer (SR) resolving any disagreements. Figure 3 outlines the flow of articles through the review process.

### Data Extraction

A template was developed in Excel to facilitate extraction of information about included articles (see Supplementary File 3). We extracted data on study characteristics, research aims, citizen scientist demographics, methods (recruitment, citizen science model, activities conducted by citizen scientists), and stakeholder engagement. Where available data on how citizen science projects were evaluated (including aims, methods and results) were extracted. One reviewer (LM) extracted the data and regularly consulted with the research team during this process.

### Collating, Summarizing, and Reporting the Results

We first undertook a quantitative descriptive analysis of included articles to report on the distribution of citizen science projects over time, by geographic location, health topic and study type (38).

To answer our first research question (*How have citizen science approaches been applied within chronic disease prevention?)* we summarized the characteristics of citizen science projects (including aims, scale, scope and longevity, recruitment methods, activities conducted by citizen scientists, and level of stakeholder engagement). To characterize the extent of citizen scientist engagement, we categorized each project according to whether it represented a contributory, collaborative, co-created or citizen-led approach (see Figure 1) in line with existing frameworks of citizen science in health (19, 20). Where available we used the original authors' classifications. Where projects adopted flexible models of engagement, allowing citizen scientists to self-select their level of involvement, we categorized the approach according to the highest level of involvement reported within the project.

To address our second research question (*How have citizen science projects been evaluated in chronic disease prevention?)*, we narratively synthesized the aims, methods, and findings of evaluations of citizen science projects.

Data synthesis was performed by the lead author (LM) and refined through ongoing discussion with the research team.

## Results

### Overview of Included Articles

**Figure 4:**
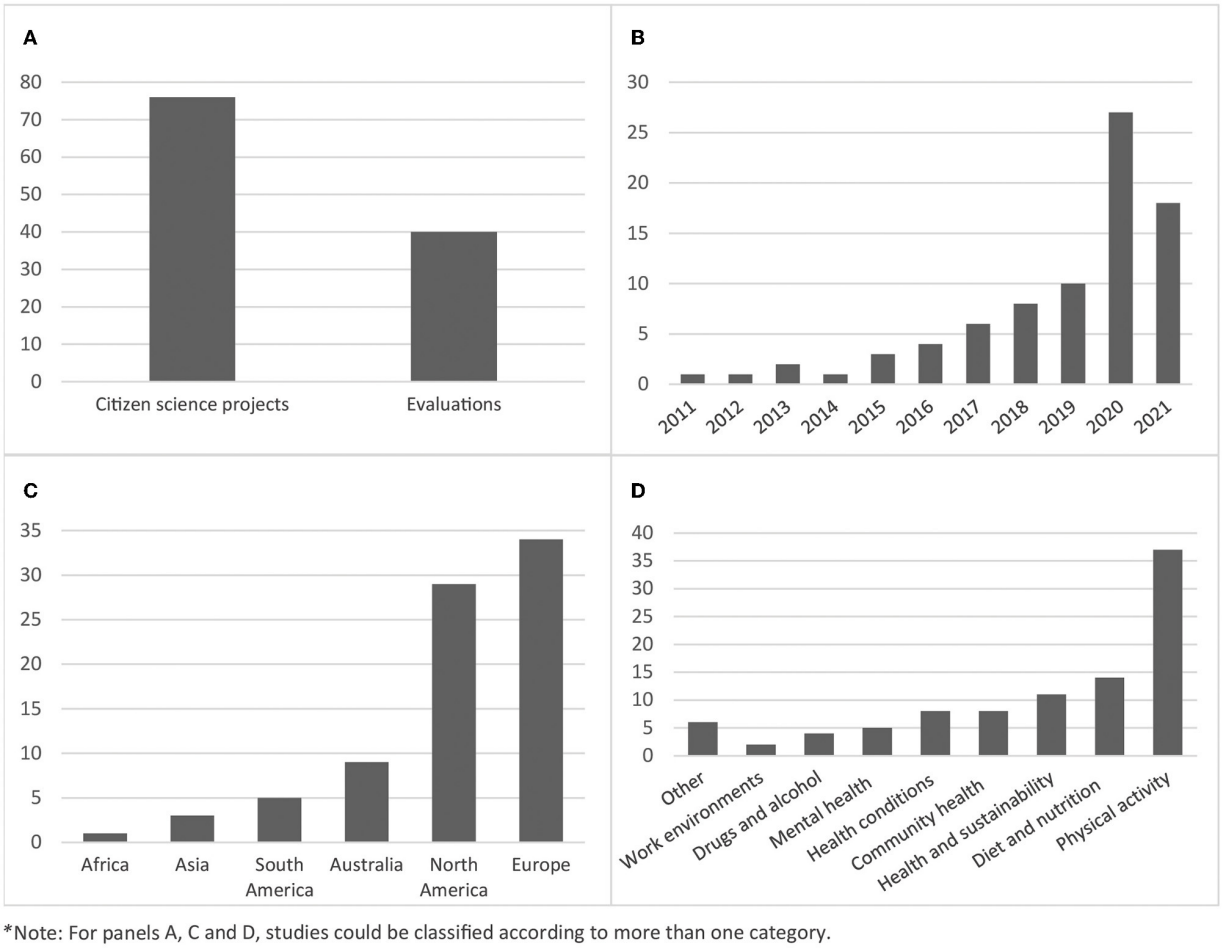
Key characteristics of included articles (*N* = 81). From top left to bottom right: **(A)** Number of articles that reported on citizen science projects or evaluations of citizen science projects; **(B)** Frequency of articles over time from 2011 to 2021; **(C)** Number of studies conducted in each continent; **(D)** Health topic area: number of studies targeting each health topic.

Eighty-one articles met the inclusion criteria for this review, of which 76 (94%) described citizen science projects in prevention, and four (5%) only reported the evaluation of a citizen science project. Of the 81 articles, 36 (44%) described both a citizen science project and its evaluation. Figure 4 summarizes the major characteristics of included articles. Further detail about each of the included articles is presented in Supplementary File 4.

The number of articles has increased exponentially since the first publication in 2011, with one third (33%) published in 2020 and one fifth (22%) in 2021. The majority of studies were conducted in the United States (*n* = 25, 31%), followed by the United Kingdom (*n* = 12, 15%), Australia (*n* = 8, 10%), Canada (*n* = 6, 7%), and Denmark (*n* = 5, 6%). Seven studies were conducted across multiple countries, and one study did not report a location and was conducted online.

Articles covered a range of health topics, with the majority focused on physical activity (*n* = 37, 46%; including active living, walkability of local neighborhoods, effects of green space on physical activity, and school-based physical activity programmes), and nutrition (*n* = 14, 17%); including access to and availability of healthy foods, farmers markets and diet. Other articles focused on health and sustainability (e.g., heat stress, community gardens/urban agriculture, oceans, and health), specific chronic health conditions (e.g., obesity, diabetes), mental health (e.g., stress, suicide prevention, effects of COVID-19), drugs and alcohol, healthy work environments or had a broader focus on community health (e.g., local barriers and enablers to health and wellbeing, age-friendly environments, neighborhood disadvantage, sexual violence prevention). Ten articles had more than one health focus.

### How Have Citizen Science Approaches Been Applied Within Chronic Disease Prevention?

Across the 76 articles reporting on citizen science projects, 73 unique citizen science projects were discussed. Data from articles reporting on the same projects were combined for the purpose of reporting. Table 1 provides an overview of the characteristics of the citizen science projects.

**Table 1:** Characteristics of citizen science projects in prevention.

**Project characteristics**	***n***	**%**
Aims of citizen science projects		
Identify problems	29	40%
Generate or prioritize solutions	21	29%
Develop or deliver intervention	21	29%
Monitor and/or evaluate interventions	20	27%
Community empowerment or capacity building	15	21%
Access novel data	11	15%
Influence health knowledge, attitudes, or behaviors	5	7%
Scale		
Small (<50 participants)	43	61%
Medium (50 to 299 participants)	11	16%
Large (> 300 participants)	16	23%
Participants' age group		
Children (<13 years)	8	13%
Adolescents (13–18 years)	18	30%
Adult (18–60 years)	46	77%
Older adults (>60 years)	35	58%
Geographic scope		
Local	44	60%
Regional	9	12%
National	8	11%
Global	12	16%
Citizen science approach		
Contributory	33	45%
Collaborative	24	33%
Co-created	15	21%
Citizen led	1	1%
Stakeholder engagement		
Project initiated or commissioned by stakeholders	5	7%
Project developed in partnership with stakeholders	14	19%
Project had limited stakeholder engagement	21	29%
No stakeholder engagement	33	45%

*Projects often had multiple aims and/or recruited participants from more than one age groups.

#### Aims of Citizen Science Projects

We identified seven key aims, with projects often having multiple aims. As shown in Table 1, 29 projects (40%) used citizen science approaches to *identify problems* from the perspective of community members. For example, the *Healthy Slotermeer* project (41), engaged local citizen scientists from a disadvantaged neighborhood in the Netherlands to carry out group interviews with community members to identify what aspects of the neighborhood residents viewed as health enhancing or hindering to health. Twenty projects used the Our Voice approach (42) across a number of countries to engage citizen scientists in identifying barriers and facilitators to healthy eating, physical activity, safety, age-friendliness or stigmatization while walking around public spaces in their neighborhood.

Twenty-one projects (29%) aimed to *generate or prioritize solutions* for policy, practice, services or research. For example, one project commissioned by a local public sector decision-making body in England engaged citizen scientists and policy makers to co-design research to inform the development of policy recommendations and priorities for interventions to reduce alcohol-related harm (43). Another project applied a citizen science approach to identify, prioritize and generate consensus on a set of policy recommendations for obesity prevention in Spain (44).

Twenty-one projects (29%) used citizen science approaches to *develop or deliver interventions*. For example, one project engaged citizen scientists in developing and implementing a community-based intervention to promote physical activity among older adults in Switzerland, including trialing the intervention in their neighborhoods, evaluating its acceptability and feasibility, and gradually taking control over organizing the program (45). A project in New Zealand engaged citizen scientists to contribute to the design of a food environments feedback system (*FoodBack*), to serve as a real-time database on indicators of the healthiness of community food environments (e.g., supermarkets, hospitals, schools) (46).

Twenty projects (27%) used citizen science approaches to *monitor and/or evaluate* prevention interventions. For example, the *MYHarvest* (47) and *Edible Gardens* (48, 49) projects used citizen science to understand experiences with urban food gardens. Other projects engaged older adults as citizen scientists in auditing public green spaces in their neighborhoods (50) and evaluating how local corner stores supported or hindered residents' access to healthy foods (51).

Fifteen projects (21%) had aims related to *community empowerment and building community capacity* to drive local change. For example, the *NESLA* project (52) used citizen science to engage and empower young males from a stigmatized neighborhood in Sweden to identify barriers and facilitators in their physical and social environments as a basis for dialogue with local decision makers about improving their neighborhoods. Another project in the United States engaged citizen scientists using Translational Advisory Boards, a representative body of 5–7 county residents to develop community-academic partnerships aimed at improving community health (53).

Eleven projects (15%) used citizen science to *access novel sources of data* to better understand a research topic area. For instance, one study engaged Danish residents online in a time-series design to understand public perspectives on the effects of the COVID-19 pandemic and related public-health measures on their mental health and behaviors (54).

Five projects (7%) used citizen science as *health and education initiatives* to influence health knowledge, attitudes, or behaviors. For example, in the *Exercise Investigation* project (55), primary school students in the United Kingdom completed a series of computer-based and outdoor educational activities to compare the acute impacts of classroom physical activity breaks with a control activity on cognition and affective wellbeing.

#### Scale, Geographic Scope, and Longevity

Citizen science projects were predominantly small-scale, with most recruiting <50 citizen scientists (see Table 1). The majority (*n* = 44, 60%) were conducted within local geographic boundaries (e.g., within a neighborhood, district or city) to address locally defined research questions and needs, with fewer projects conducted at a regional, national or global scale. One-off and ‘pilot’ projects were most frequent (*n* = 49, 67%), but a third of projects (*n* = 24, 33%) were (or were intended to be) long running or scaled up to other settings.

#### Recruitment of Citizen Scientists

Sixty-five projects reported on recruitment of citizen scientists. Larger-scale projects often recruited citizen scientists via online channels such as project websites and social media, and/or via mass media and email mailing lists, while smaller-scale projects tended to recruit citizen scientists through local community channels. Common sampling approaches for small and medium scale projects included snowball sampling through community champions, dissemination of study information at local community events, via community-based and partner organizations (such as schools, youth centers, senior centers), advertisements distributed in public spaces (e.g., shops, billboards or mailboxes), mass media channels, online via social media or project websites. Twenty-one projects (32%) reported providing incentives or renumeration for participation, including small financial payments, gift cards, refreshments, or merchandise.

Of the 60 projects which provided demographic information on their participants age the majority (*n* = 46, 77%) involved adults (aged 18–60 years) and/or older adults (>60 years, *n* = 35, 58%) (see Table 1). In almost half of the 58 projects which reported participants' gender (*n* = 28, 48%) more than 65% of citizen scientists were female, including two projects which purposively recruited all women. Twenty-one projects (29%) purposively recruited citizen scientists from specific population groups based on other demographic characteristics such as those living in low-income or rural areas, cultural and ethnic minority groups, or people living with overweight/obesity or chronic disease. For example, Zieff et al. (56) recruited citizen scientists, including adults experiencing homelessness, in three socioeconomically diverse cities in Mexico, Chile and the United States, to gather perceptions of neighborhood characteristics or infrastructure that support or impede physical activity.

#### Activities Performed by Citizen Scientists

**Figure 5:**
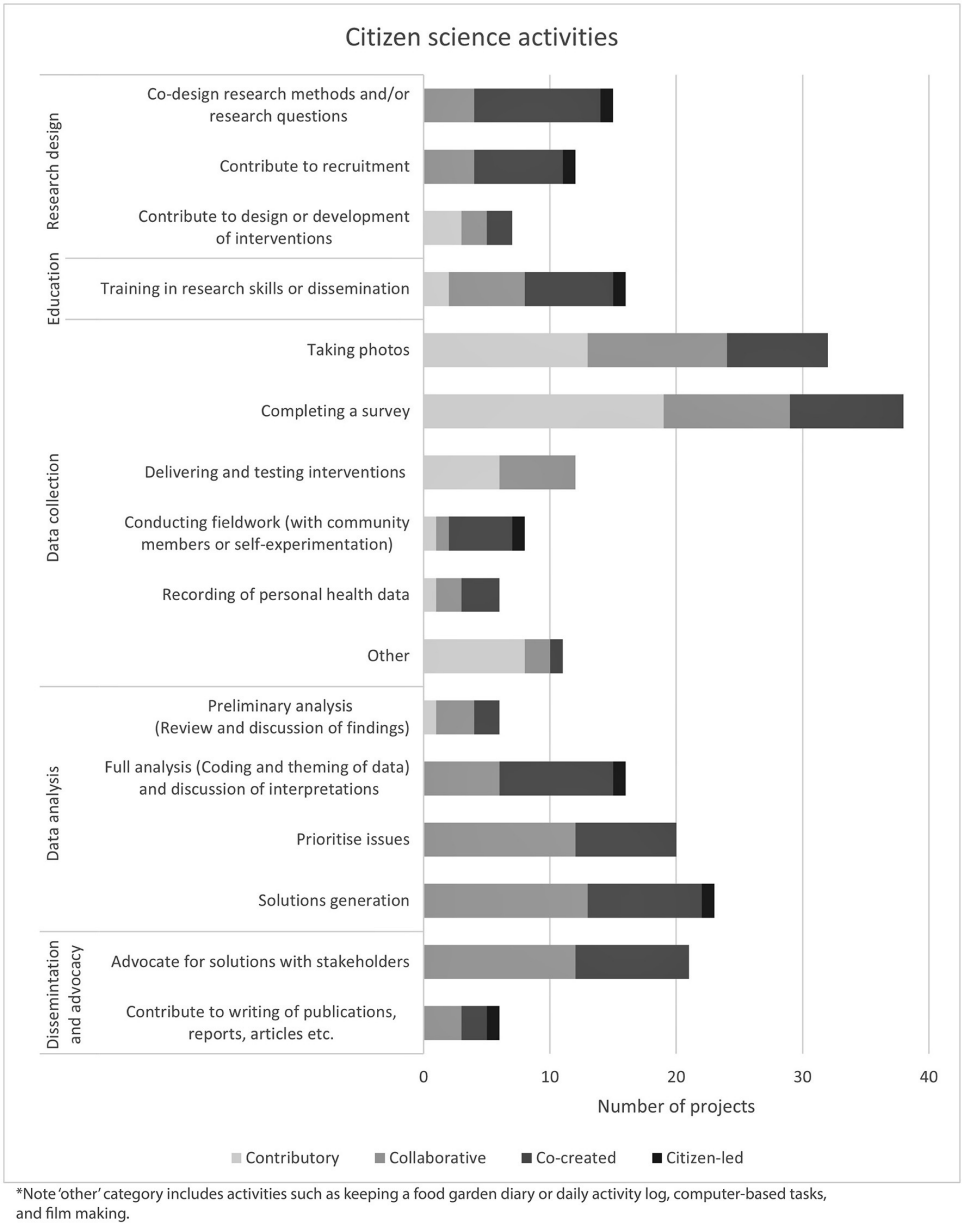
Breakdown of activities performed by citizen scientists in citizen science projects (*n* = 73).

A summary of activities performed by citizen scientists across the different phases of the research process is provided in Figure 5. Small, local projects frequently used more intensive face-to-face data collection, analysis and solution generation approaches. In a number of these projects' citizen scientists worked with researchers to interpret their findings, draw conclusions or develop recommendations, and subsequently disseminate findings and/or advocate for their recommendations and proposed solutions with local policy makers (e.g., in councils or government departments); this usually took place in community meetings, facilitated by researchers. For example, one study engaged members of a senior citizens center in Australia to identify features of their physical environment that facilitate or hinder walkability or access to the center and brought citizen scientists together to analyse their data, prioritize recommendations and advocate for change (57). In contrast, larger scale projects predominantly used online data collection methods (e.g., through apps and online surveys) and rarely involved participants beyond the data collection phase. However, one exception, was *The Healthy Communities project* in a village in England (58), in which residents were trained to collect data on local issues related to healthy eating and physical activity, and co-produce and implement local solutions to enable healthier lifestyles with community organizations. Although only a handful of community members were involved in the beginning stages, participation grew from 4 to 4,000 participants over the course of the 7-month project.

Sixteen projects (22%) provided education for citizen scientists, which often took the form of research skills training workshops. Topics included developing research questions, methods and study design, collecting data in an ethical and rigorous manner, analysis, and advocacy training. Training of citizen scientists to conduct fieldwork activities such as participant interviews was a notable feature of four projects (41, 43, 59, 60).

#### Citizen Science Approach

When considering the level of engagement of citizen scientists (see Figure 1), most projects adopted a contributory or collaborative citizen science approach, with fewer projects involving citizen scientists as research partners or lead investigators in co-created or citizen-led approaches. A summary of projects according to each of the citizen science models is provided below, with examples of each approach in Box 1.

Box 1 **Example studies.**

**Contributory model**

One study sought to explore the feasibility of using citizen science to gather data on community-level physical activity (61). Sixty-one citizen scientists observed people engaging in physical activity at five local greenways and recorded demographics (estimated age and race), activity (walking, running, or bicycling) and intensity level (moderate, vigorous) for each individual they observed using validated observation forms. Citizen scientists uploaded their observations via an online portal.

**Collaborative model**

The FEAST study (62) used the Our Voice approach to identify factors that influence older adults' ability to access, choose, and buy healthy food, and to prioritize and advocate for the concerns of older adults related to choosing and buying food. Twenty-three citizen scientists used an app to collect photos and audio narratives of features of food stores that impacted their ability to choose and buy healthful foods. Eleven of the citizen scientists subsequently came together in 2 community meetings, facilitated by researchers, to review and discuss their findings, prioritize issues to address and present issues to key stakeholders and local policy makers.

**Co-created model**

One study engaged youth of the Karuk Tribe in California in the co-creation of research to assess community health, access to healthy food and physical activity in order to inform the Tribe's prioritization of services and resources (59). Twelve adolescent citizen scientists undertook research and leadership training workshops to develop skills to conduct research in an area of healthy food and physical activity of interest to them, in which they formulated research questions, study design and methods, conducted their research with members of the local community, analyzed the data together with researchers to discuss possible interpretations and findings, and subsequently generated recommendations to present to the Tribe's decision makers.

**Citizen-led model**

Citizen scientists in the blood testers project (63) - a participant-led research (PLR) project of blood lipids - determined hypothesis and research questions of personal interest to them and carried out single-subject experiments, documenting their data, and discussing their findings in online groups of participating citizen scientists. Throughout the study, citizen scientists collaboratively identified and generated a list of risks and risk mitigation strategies for participation to inform draft ethical review and governance practices for ongoing PLR projects.

##### Contributory Approaches

A contributory citizen science approach was most common overall (*n* = 33, 45%), predominantly involving citizen scientists only in data collection activities, such as conducting observations or audits (e.g., via photos, audio narratives, surveys or diary entries), or in basic analysis activities such as group discussions of the data. The time citizen scientists spent participating in contributory projects ranged from one-off activities of up to 1 h, to a series of structured activities over the space of a day, weeks, or months.

##### Collaborative Approaches

A third of projects used a collaborative approach (*n* = 24, 33%), which typically involved citizen scientists working with researchers in collecting, classifying, synthesizing, and interpreting data, brainstorming or prioritizing potential solutions, and/or disseminating findings and advocating for change. Citizen scientists' involvement in collaborative projects frequently took place over multiple sessions, spanning weeks, or months.

##### Co-created Approaches

A fifth of projects adopted a co-created approach (*n* = 15, 21%), in which researchers worked in partnership with citizen scientists over longer periods of time to co-design many or all stages of the project. Co-created projects often took community-based participatory research, or action research approaches, with a focus on working closely with community members, especially those from disadvantaged or high-risk communities to identify problems (i.e., community health and wellbeing needs) and advocate for solutions. Citizen scientists were commonly involved from the outset and carried out more complex tasks, such as formulating or refining research questions, methodologies and data collection instruments, recruiting other citizen scientists or research participants and carrying out fieldwork activities (including conducting questionnaires, interviews or focus groups with participants). Due to the nature of these activities, participation in co-created projects tended to occur across multiple research stages and involve greater time commitments often lasting several months or years (from 2 to 24 months).

##### Citizen-Led Approaches

One project was classified as citizen-led (2%), with the research process, including design and execution, led entirely by citizen scientists, who developed their own research questions and carried out single-subject experiments or “N-of-1” research (63).

#### Policy and Practice Stakeholder Engagement

Over half of the citizen science projects (*n* = 40, 55%) engaged key stakeholders such as practitioners, policy makers, or community organizations, in addition to researchers and citizen scientists. Of these, 21 projects (29%) had limited stakeholder engagement, primarily including reporting findings to stakeholders at the end of projects. While 19 projects actively involved these stakeholders through the research process, including 5 projects (7%) which were initiated or commissioned by stakeholders from their outset, and 14 projects (19%) which were developed and conducted in partnership with stakeholders. In these projects, stakeholders were engaged in various aspects of the research process, including informing study design, recruiting citizen scientists, and translation of findings into practice. One example was a partnership project between the Northern Kentucky Health Department and a community health organization who worked with citizen scientists to collect data, educate the community, build community support, and advocate for and pass a local smoke-free policy in a rural, low SES community in Kentucky (64).

### How Have Citizen Science Projects in Chronic Disease Prevention Been Evaluated?

Forty articles (49%) reported on evaluations of citizen science projects. Twenty-two projects (55%) conducted a formal evaluation and reported the methods used and findings. The remaining eighteen projects (45%) did not report any formal evaluation methods but reflected on the process of implementing citizen science approaches and/or reported on overall project impacts. Evaluation data were typically gathered using pre- and post- surveys or questionnaires, interviews and/or focus groups, with observation and weekly meetings with citizen scientists used in two projects.

#### Evaluation of Process Elements

Twenty-three projects (58%) included process evaluation, examining motivations for and experiences of participating in citizen science projects, and/or the feasibility or utility of citizen science approaches.

##### Motivation to Engage in Citizen Science Projects

Six projects (15%) examined the motivations of citizen scientists. Motivations included personal health and wellbeing benefits, an interest in the health topic, enhancing social connectedness and the opportunity to meet new people, contributing to a cause (e.g., improving one's neighborhood or making a difference in the community) and learning new skills (e.g., in citizen science and research, personal development). Two projects investigated the influence of rewards on motivation to participate, with findings from one project indicating that some citizen scientists were motivated by financial incentives (41), while another by educational course credits (61). One project found that time constraints and the use of the term “citizen science” were barriers to participation (65).

##### Experiences of Participating

Eight projects (20%) examined experiences of participating in citizen science projects. All indicated that citizen scientists were able to successfully and confidently complete research activities, and three projects also indicated that citizen scientists had positive experiences throughout the research process, and in some cases expressed interest in participating in future citizen science projects. Citizen scientists in three projects also provided feedback on key challenges they encountered through participating and suggestions for improvements. These included a need to ensure the data collection process is simple and efficient (e.g., electronic as opposed to paper-based audit forms) (45, 66), and preferences for providing a greater variety of roles for citizen scientists across the whole research process, including involving citizen scientists earlier on in projects (e.g., in co-designing the research methods) (50).

##### Feasibility and Utility of Citizen Science Approaches

Seventeen projects (43%) considered the feasibility and/or utility of citizen science approaches, mainly from the perspectives of researchers, although two projects empirically examined these aspects and reported evaluation methods. One notable example was a study which evaluated the utility of adding the Our Voice engagement model, to a Safe Routes to School (SRTS) program—a national US program promoting safe options for walking or biking to school, and found the addition of using a citizen science approach increased students' engagement with the program, and was associated with higher rates of walking/biking to school compared to SRTS alone (67). Overall reflections by researchers suggested citizen science approaches offer feasible and useful ways to: (1) generate rich data that contribute unique perspectives and insights and/or may be otherwise inaccessible beyond the scope of a small university-based research team alone; (2) identify and advocate for diverse “community-focussed” solutions (68); (3) to catalyze community action to promote community health and wellbeing and address health disparities; and (4) to develop new partnerships or collaborations for ongoing work. Key factors highlighted as contributing to the success of projects included relationships developed with policy and practice stakeholders and community partners and working with community champions to facilitate recruitment of citizen scientists and increase potential project sustainability. Across projects several challenges associated with using citizen science approaches were also identified, including difficulties recruiting sufficiently large or diverse samples of citizen scientists, burden on citizen scientists (e.g., time and energy commitments) and their capacity (e.g., time and research skill) to complete certain activities in line with traditional systematic research protocols. Technological constraints (e.g., internet inequity or using mobile sensor-based technologies for data collection), and resourcing constraints (e.g., inadequate time or funding) for more intensive, longer-term projects and follow-up were also cited as key challenges.

#### Impacts of Citizen Science Projects

Thirty projects (75%) reported on impacts arising from citizen science projects, including benefits for citizen scientists and policy and practice. Three projects utilized an existing evaluation framework (41, 69–71), to assess potential benefits of participating in citizen science projects. Short-term impacts of citizen science projects were most often reported, although one study documented impacts at 3, 6, 12, and 24 months follow up and found benefits at the individual, community, and population levels (62).

##### Impacts on Citizen Scientists

Twenty-two projects (55%) reported impacts of participation on the citizen scientists, including benefits and/or unintended consequences of participation. Impacts included improvements: in scientific literacy (e.g., greater skills and confidence in designing and conducting research); health literacy (e.g., increased knowledge about the topic area or their own health); lifestyle behaviors (e.g., healthier eating patterns, increased physical activity); empowerment and capacity for action (e.g., ownership over project, greater self-efficacy to take action); and social connectedness (e.g., expanded social networks, social skills, sense of community).

##### Impacts on Policy and Practice

Eighteen projects (45%) reported policy or practice impacts arising from citizen science projects. Seventeen of these were projects that had been developed in collaboration with policy and practice stakeholders and community organizations. Impacts included: successful policy implementation or evidence being used to inform policies, priorities, strategic planning and resource allocation; the development of new programs, committees or grant funding to address locally identified needs; and implementation of community recommendations to promote healthy behaviors and/or create healthier environments (e.g., a community garden, gardening lessons, cooking lessons, a food security program for children, additional bike racks in schools, street signage and footpath repairs to promote safe physical activity etc.). For example, in one study citizen scientists identified a set of priorities and recommendations for alcohol-related harm interventions, which were accepted for action by the commissioning policy makers, and informed further investment in community-based research to explore healthy eating policies (43). Another project found taking a citizen science approach built community interest and support for a local physical activity program, resulting in its refunding and scale up, and strengthened relationships between community members, policymakers and academic researchers (72). Authors of one study also reflected on an improved relationship between community and local government as a result of working together through the citizen science process (58). Five projects also reported anticipated outcomes, where efforts had been made to inform or influence policy changes but had not yet been realized at the time of publishing.

## Discussion

### How Have Citizen Science Approaches Been Applied Within Chronic Disease Prevention?

Citizen science approaches show considerable potential as a means of engaging the public in gathering and making sense of data, identifying the drivers of health problems and contributing to the development of effective and acceptable solutions in prevention. In line with a growth in the use of citizen science approaches across disciplines over the past decade (23, 73), our findings show a considerable increase in the application of citizen science in chronic disease prevention, suggesting these approaches are rapidly gaining traction in this field, especially in the areas of physical activity and nutrition. This focus likely speaks to the relative ease of using citizen science approaches to capture data on physical environments, for example taking photographs or completing surveys on features that support or hinder physical activity and healthy eating. While there were examples of projects that focused on other areas such as tackling alcohol-related harm (43), online alcohol advertising (74), and tobacco prevention policy advocacy (64), it appears that there is considerable opportunity to broaden the scope of citizen science in prevention.

Within citizen science projects there is often a trade-off between the depth of citizen scientist engagement and the scale and scope of the project. This was illustrated within our findings, with only 39% of projects recruiting more than 50 citizen scientists and these larger-scale projects typically involving citizen scientists in a “contributory” fashion, usually as data collectors. By contrast local, smaller scale projects tended to take a more “collaborative” approach, providing opportunities for citizen scientists to be involved in other elements of the research process, including design, data analysis and interpretation and dissemination and advocacy activities. This attention to meaningful participation of smaller numbers of citizen scientists likely stems from the tradition of community-based participatory research approaches in public health, in which community members are valued as equal partners in the research process (75). However, these findings suggest there is untapped potential to apply the kinds of large-scale and collaborative citizen science approaches demonstrated in other disciplines to be powerful tools for community engagement, advocacy, and education (76–80). For example, large-scale approaches may be particularly beneficial in prevention, for gathering data that allows for monitoring and evaluation of policies and programs not just at local but also state, national and international levels. Work by Okop et al. (81) and Katapally et al. (82, 83) provide examples of how larger scale citizen science projects in prevention can be implemented across multiple communities and/or countries to generate rich, population-level data to inform decision making. The past decade has seen increasing attention to the development and testing of a range of innovative methods and tools to engage people in large scale citizen science projects, particularly using mobile apps and gaming approaches (46, 83–89), which we should capitalize on in chronic disease prevention.

Although 29% of citizen projects identified in this review deliberately targeted specific population groups to address issues of concern (e.g., low-income, public housing residents, ethnic minority groups), we found that outside of these targeted projects there was a lack of attention to ensuring diversity and inclusion in terms of culture, gender, and socio-economic status. For example, a fifth of projects (21%) did not report citizen scientists' gender, and over half (56%) did not report other pertinent sociodemographic information (e.g., race, ethnicity, education and socio-economic status). Engaging diverse perspectives is critical to addressing inequities in population health, and citizen science offers opportunities to increase participation of population groups typically excluded from research and decision-making processes (59, 90). It is important for citizen science projects to consider and report on their strategies for ensuring diversity and inclusivity.

The majority of citizen science projects identified within this review have been one-off and short in duration, which is resource intensive to set up, and in some cases represents missed opportunities for fostering long-term engagement and relationship building between citizen scientists, communities and other stakeholders involved. Prior research suggests long-running citizen science projects are more likely to be cost-effective (91), but currently there are inadequate funding mechanisms and infrastructure to enable longevity and scalability. One avenue to explore is how citizen science approaches may be supported and embedded within the practice of governments and other key organizations as an additional tool within their “toolbox” to generate data to underpin policy and practice, and Roger et al. (92) provide an example of this. If citizen science is to be more widely adopted and supported in prevention, and done in a sustainable and cost-effective way, we will need to reconsider how these projects are funded to go beyond one-off, time-limited project funding (93).

While half of the citizen science projects in our review involved some level of stakeholder involvement, only a quarter were led by policy or practice stakeholders or involved them in core roles, suggesting there is considerable potential to better engage these knowledge users in citizen science projects. Ensuring collaboration and involvement of policy and practice stakeholders is built into citizen science projects, from an early stage, is crucial to maximizing the capacity for translation of findings into policy and practice (93, 94). Such community-researcher-stakeholder collaborations offer much promise for co-creating actionable research evidence and developing shared agendas that reflect communities' perspectives and needs. However, to date there has been little consideration of stakeholders' perceptions of the relevance and value of citizen science approaches, and this is the focus of some of our current work (95).

### How Have Citizen Science Projects Been Evaluated in Chronic Disease Prevention?

Our findings demonstrate that citizen science projects have begun to realize impacts that align with prevention goals, including improving health literacy, empowering communities and building community capacity to support and advocate for actions and bringing about community-driven environmental and policy changes to promote health and wellbeing and create healthier environments. However, only 49% of studies included an evaluation component, half of which reported their evaluation methods and findings, highlighting the need for more robust evaluation to assess the feasibility, delivery and/or impacts of citizen science approaches. Evaluations of longer-term impacts were especially scarce, with most evaluations focussed on reporting short-term project outcomes. This limits our ability to draw general insights about what factors contribute to the development of successful projects and how project impacts are brought about and for whom. Evaluation frameworks [such as those developed by Kieslinger et al. (96) or Den Broeder et al. (19)] could be more widely applied to capture robust evaluation data and enable citizen science practitioners and stakeholders to build vital knowledge to guide the design, implementation and evaluation of citizen science in prevention. Additionally, as understanding of how to design, deliver and evaluate citizen science projects grows, an emerging need is to better develop and share best practices, protocols and instruments to assess citizen science initiatives and facilitate replication and scale-up in other settings (97).

### Limitations

Due to a lack of consensus in the field as to what constitutes citizen science (24, 98) and ongoing debate around appropriate terminology (15), we made a pragmatic decision to only include studies which explicitly defined their approach as “citizen science.” As such, studies which used approaches akin to citizen science but did not explicitly use the term “citizen science” would not have been identified by our search strategy. Broadening the criteria to include studies that use public engagement approaches but do not explicitly refer to themselves as citizen science would, however, open up myriad questions about where to draw the boundaries of these approaches.

Secondly, our analysis of the characteristics and evaluations of citizen science projects was limited by the quality of reporting by studies, with several inconsistencies in reporting of citizen science methods and findings encountered across articles. We may have inadvertently missed aspects of citizen science projects or evaluation findings if these were not reported in included articles or due to a lack of clarity in reporting. This highlights the need for improvements and consistency in reporting of health-related citizen science projects.

Finally, in line with methodological guidance for conducting and reporting scoping reviews (99, 100), we did not conduct quality appraisal of included studies in this review. Due to the infancy of this field and lack of frameworks for reporting on citizen science studies in the health and biomedical sciences we did not consider quality assessment of included studies appropriate in this review.

## Conclusion

Engaging the public in chronic disease prevention is crucial if we are to develop pragmatic, equitable and acceptable solutions to the complex public health challenges we face, and citizen science approaches provide innovative and engaging ways to meaningfully involve the public in research and decision making to promote health and wellbeing. This scoping review shows that research on citizen science in chronic disease prevention is a rapidly emerging area and offers promise to identify issues of relevance to communities' experience of health and wellbeing, generate and prioritize community-focussed solutions, and build community capacity and partnerships to drive change, across a wide range of health issues. While citizen science is increasingly being utilized in prevention, in order to realize the potential of this approach, and support its uptake among the diverse stakeholders that stand to benefit from its use, more attention needs to be paid to capturing and maximizing its impacts, and to evaluating the feasibility of using citizen science approaches at scale. Indeed, globally there is growing investment of time, effort and resources into supporting the use of citizen science approaches and translating findings into impacts on research, policy and practice. The use of citizen science approaches in chronic disease prevention thus rests upon whether and how these approaches are embedded within broader, sustainable strategies for public engagement in research, policy and practice in prevention.

## Author Contributions

LM, SR, and YL conceptualized the study, collaborated in developing the inclusion/exclusion criteria, conducting article screening, data analysis, and drafting of the manuscript. LM lead the development of the manuscript. YL directed the development of the search strategy and implemented the search in the databases. HT and BS edited continuous iterations of the manuscript draft. All authors contributed to the article and approved the submitted version.

## Funding

This research was supported by the Australian Prevention Partnership Centre through the NHMRC partnership centre grant scheme (Grant ID: GNT9100003) with the Australian Government Department of Health, ACT Health, Cancer Council Australia, NSW Ministry of Health, Wellbeing SA, Tasmanian Department of Health, and VicHealth. It is administered by the Sax Institute.

## Conflict of Interest

The authors declare that the research was conducted in the absence of any commercial or financial relationships that could be construed as a potential conflict of interest.

## Publisher's Note

All claims expressed in this article are solely those of the authors and do not necessarily represent those of their affiliated organizations, or those of the publisher, the editors and the reviewers. Any product that may be evaluated in this article, or claim that may be made by its manufacturer, is not guaranteed or endorsed by the publisher.
