# Supplementary material for: A Scoping Review of Citizen Science Approaches in Chronic Disease Prevention
Source: Front Public Health. 2022 May 9;10:743348. doi: 10.3389/fpubh.2022.743348 (PMC9125037; doi:10.3389/fpubh.2022.743348)
Supplement: Supplementary file 1 [file Data_Sheet_1.pdf]

Supplementary File 1: PRISMA-ScR checklist

Preferred Reporting Items for Systematic reviews and Meta-Analyses extension for Scoping Reviews (PRISMA-ScR) Checklist[1]

SECTION	ITEM	PRISMA-ScR CHECKLIST ITEM	REPORTED ON PAGE #
**TITLE**			
Title	1	Identify the report as a scoping review.	Page 1
**ABSTRACT**			
Structured summary	2	Provide a structured summary that includes (as applicable): background, objectives, eligibility criteria, sources of evidence, charting methods, results, and conclusions that relate to the review questions and objectives.	Page 1
**INTRODUCTION**			
Rationale	3	Describe the rationale for the review in the context of what is already known. Explain why the review questions/objectives lend themselves to a scoping review approach.	Page 2
Objectives	4	Provide an explicit statement of the questions and objectives being addressed with reference to their key elements (e.g., population or participants, concepts, and context) or other relevant key elements used to conceptualize the review questions and/or objectives.	Page 2
**METHODS**			
Protocol and registration	5	Indicate whether a review protocol exists; state if and where it can be accessed (e.g., a Web address); and if available, provide registration information, including the registration number.	N/A
Eligibility criteria	6	Specify characteristics of the sources of evidence used as eligibility criteria (e.g., years considered, language, and publication status), and provide a rationale.	Page 4
Information sources*	7	Describe all information sources in the search (e.g., databases with dates of coverage and contact with authors to identify additional sources), as well as the date the most recent search was executed.	Page 2
Search	8	Present the full electronic search strategy for at least 1 database, including any limits used, such that it could be repeated.	Supplementary materials page 3
Selection of sources of evidence†	9	State the process for selecting sources of evidence (i.e., screening and eligibility) included in the scoping review.	Page 4
Data charting process‡	10	Describe the methods of charting data from the included sources of evidence (e.g., calibrated forms or forms that have been tested by the team before their use, and whether data charting was done independently or in duplicate) and any processes for obtaining and confirming data from investigators.	Page 4
Data items	11	List and define all variables for which data were sought and any assumptions and simplifications made.	Supplementary materials page 4
Critical appraisal of individual sources of evidence§	12	If done, provide a rationale for conducting a critical appraisal of included sources of evidence; describe the methods used and how this information was used in any data synthesis (if appropriate).	N/A
Synthesis of results	13	Describe the methods of handling and summarizing the data that were charted.	Page 4
**RESULTS**			
Selection of sources of evidence	14	Give numbers of sources of evidence screened, assessed for eligibility, and included in the review, with reasons for exclusions at each stage, ideally using a flow diagram.	Figure 3, Page 5

SECTION	ITEM	PRISMA-ScR CHECKLIST ITEM	REPORTED ON PAGE #
Characteristics of sources of evidence	15	For each source of evidence, present characteristics for which data were charted and provide the citations.	Supplementary materials page 5
Critical appraisal within sources of evidence	16	If done, present data on critical appraisal of included sources of evidence (see item 12).	N/A
Results of individual sources of evidence	17	For each included source of evidence, present the relevant data that were charted that relate to the review questions and objectives.	N/A
Synthesis of results	18	Summarize and/or present the charting results as they relate to the review questions and objectives.	Page 4-11
**DISCUSSION**			
Summary of evidence	19	Summarize the main results (including an overview of concepts, themes, and types of evidence available), link to the review questions and objectives, and consider the relevance to key groups.	Page 11
Limitations	20	Discuss the limitations of the scoping review process.	Page 12
Conclusions	21	Provide a general interpretation of the results with respect to the review questions and objectives, as well as potential implications and/or next steps.	Page 12
**FUNDING**			
Funding	22	Describe sources of funding for the included sources of evidence, as well as sources of funding for the scoping review. Describe the role of the funders of the scoping review.	Page 13

Supplementary File 2: Search terms

**Search terms**	(“citizen scienc*”)<br>AND<br>(health* OR lifestyle* OR prevent* OR diet* OR food* OR obes* OR "physical* activ*" OR smok* OR tobacco OR nutrition* OR alcohol* OR transport* OR "active commuting" OR "urban design" OR "liveability" OR “livability” OR "urban planning" OR walk* OR social* OR wellbeing)

Supplementary File 3: Data extraction template

Field	Notes
**Author**	[Names of authors]
**Year**	[Year published]
**Title**	[Publication title]
**Journal**	[Journal name]
**Country**	[Country in which the study was conducted]<br>(If multiple countries for data collection then list all here)
**Project name**	[Name of project, if specified]
**Topic/focus**	[Prevention topic the study was targeted to address]<br>(e.g. physical activity, food environments, diet and nutrition, community health, built environments etc.)
**Article type**	[Focus of the citizen science approach used e.g. to report on a citizen science project or an evaluation of a citizen science project]
**Brief project description/aims**	[Research questions, study aims and objectives]
**Longevity of project/activities**	[Duration of study and/or citizen science activities]
**Participants**	[#, age, sex, pertinent characteristics of citizen scientists]
**Recruitment**	[Recruitment methods for engaging citizen scientists, and any renumeration provided]
**Policy or practice engagement**	[Describe any policy or practice stakeholder engagement or collaboration]
**What activities do citizen scientists do?**	[Key activities citizen scientists conducted] (e.g. data collection, analysis, advocacy, dissemination, training).
**Citizen science model**	Approach to citizen engagement adopted, if specified e.g., contributory collaborative, co-created or citizen-led]
**Findings**	[Key findings reported e.g. from citizen science projects and evaluations of citizen science projects]
**Evaluation aims**	[Aims and objectives of evaluation, if specified]
**Evaluation methods**	[Evaluation methods, if specified]
**Evaluation findings**	[Evaluation findings, if reported including motivations to engage, experiences of participating, feasibility or utility of citizen science approaches, and key limitations or challenges of projects, where related to the citizen science approach taken]
**Impacts on citizens**	[Reported impacts of projects upon citizen scientists, if specified]<br>(e.g. scientific literacy, health literacy, social skills, empowerment, research skills)
**Impacts on policy or practice**	[Reported impacts of projects on policy or practice, if specified] e.g.

Supplementary File 4: Overview of articles reporting on citizen science projects and evaluations in prevention (N = 81)

Author (Year)	Prevention topic	Article type		Citizen scientists (#, characteristics)	Citizen science model	Research activities citizen scientists were involved in				Policy or practice engagement	Reports on impacts	
		Citizen science project	Evaluation			Research design	Data collection	Data analysis	Dissemination and advocacy		Policy or practice	Citizen scientists
Abu et al[2]	Health and sustainablilty	X	X	n/a	Contributory		X					
Agostini et al[3]	Health conditions (Obesity)	X	X	10 citizen scientists (aged 23–60 years, 60% female)	Collaborative		X	X				X
Andersen et al[4]	Other (Sleep quality and smartphone use)		X	8,894 citizen scientists (aged 16+ years, 61% female)	-	-	-	-	-			X
Armstrong et al[5]	Health and sustainablilty (sustainable diets)	X		516 citizen scientists (characteristics n/a)	Contributory		X					
Armstrong et al[6]	Health and sustainablilty (sustainable diets)	X	X	601 citizen scientists (characteristics n/a)	Contributory		X					
Barrie et al[7]	Physical activity (Green space)	X	X	15 citizen scientists (aged 60-84 years, 80% female, retired, some lived in rural towns)	Co-created	X	X	X				X
Booth et al[8]	Physical activity (School-based physical activity programmes)	X		5463 citizen scientists (aged 9-10 years, 51% female, primary school students)	Contributory		X					
Buman et al[9]	Diet and nutrition (Farmers' markets)	X		38 citizen scientists (aged 18-35 years, 65% female)	Contributory		X					
Buman et al[10]	Diet and nutrition (Food environments); and Physical activity (Built environments)	X	X	9-12 citizen scientists (aged 65+ years, low-income, living in public housing)	Co-created		X	X	X	X		X
Buman et al[11]	Physical activity (Built environments)	X		27 citizen scientists (aged 65-90 years, 73% female, low-income, members of	Contributory		X			X		

				racial and ethnic-minority groups)								
Burcham et al[12]	Diet and nutrition and Health conditions (Oral Health)	X		351 citizen scientists (including 172 adults aged 20-75 years and 179 youth aged 7-16 years)	Contributory		X	X				
Cambria et al[13]	Physical activity (Green space)	X		n/a	Contributory		X					
Cervinka et al[14]	Physical activity (green space) and Mental health	X		99 citizen scientists (aged 16-81 years, 64% females)	Co-created	X	X	X	X	X		
Chesser et al[15]	Community health (age-friendly environments)	X		10 citizen scientists (aged 68-78 years, 80% female, university students, staff and community members)	Collaborative		X	X	X	X		
Chrisinger and King[16]	Mental health (chronic stress and wellbeing)	X		14 citizen scientists (age n/a, 57% female)	Contributory		X			X		
Chrisinger et al[17]	Diet and nutrition (Food access)	X	X	8 citizen scientists (aged 18–40 years, 50% female, members of racial minority groups)	Collaborative		X	X	X	X	X	X
Clotworthy et al[18]	Mental health (COVID-19 effects)	X		11,494 citizen scientists (aged 18-87 years, including some families with children living at home and older people)	Contributory		X					
Collins et al[19]	Other (Breastmilk composition, breastfeeding journeys)	X	X	722 citizen scientists (aged 25–44 years, > 95% female, parents to 1 or 2 children)	Co-created	X	X	X				X
De Cocker et al[20]	Physical activity (Sedentary behaviour)	X	X	6,246 participants (aged 37.5 years on average, 65.8% female)	Contributory		X					
Den Broeder et al[21]	Community health	X	X	35 citizen scientists (aged 21-60+ years, 91% female, low SES, members of cultural minority groups)	Collaborative	X	X	X	X	X	X	X
Díaz et al[22]	Health conditions (mHealth for chronic condition self-management)	X	X	22 citizen scientists (aged 37-55 years, 68% female)	Collaborative	X	X			X		X

Díez et al[23]	Diet and nutrition (Food environments); and Physical activity (Built environments)	X	X	36 citizen scientists (aged 31-72 years, 50% female; many low-income)	Collaborative		X	X	X	X		X
Dilley et al[24]	Physical activity (Cycling safety)	X	X	61 citizen scientists (aged 15-79 years, white and African American)	Contributory		X					
Dissing et al[25]	Other (Sleep quality and smartphone use)	X		24,856 citizen scientists (aged ≥16 years, 62% female)	Contributory		X					
Dobson et al[26]	Health and sustainability (Urban agriculture)	X		163 citizen scientists (characteristics n/a)	Contributory		X					
Dolejšová and Kera[27]	Diet and nutrition (Soylent Diet)		X	43 citizen scientists (aged 25-35 years, 14% female)	-	-	-	-	-			X
Eames and Egmose[28]	Health and sustainability	X		34 citizen scientists (aged 16-65+ years, 68% women, including 3 participant panel groups: young people (*n*=12); women (*n*=11); and older people (*n*=11)	Collaborative		X	X	X	X		
Edmondson et al[29]	Health and sustainability (Urban agriculture)	X		n/a	Contributory		X					
Fancourt, Baxter, and Lorencatto[30]	Mental health (Depression and Anxiety)	X		3,532 citizen scientists (aged 18-65+ years, 58.2% female, some with anxiety, and/or depression)	Contributory		X					
Fell et al[31]	Community health (Neighbourhood stigmatization)	X	X	23 citizen scientists (aged 16-18 years, 10% male, residing in a disadvantaged neighbourhood)	Co-created		X	X		X	X	X
Folkerth et al[32]	Drugs and alcohol (Tobacco prevention policy)	X	X	n/a	Collaborative		X		X	X	X	
Frei et al[33]	Physical activity	X	X	29 citizen scientists (aged 61-91 years, 76% female, average BMI 27.8 (± 4.1)	Collaborative	X	X			X		X
González et al[34]	Diet and nutrition (Food environments);	X	X	97 citizen scientists (including 39 children aged 9-12 years, and 58	Co-created		X	X	X	X	X	

	and Physical activity (Built environments)			adolescents aged 13–18 years), 65% female, 60% living in low SES neighbourhoods)								
Graham et al[35]	Community health (Sexual violence prevention)	X		10 citizen scientists (age n/a, 100% female, undergraduate students, mixed race/ethnicity groups, mix of sexual orientations)	Co-created	X	X	X				
Grant, Wolf, and Nebeker[36]	Other (Ethical review of citizen-led research on blood lipids)	X	X	24 citizen scientists (aged 22-70 years, 25% female, 23% had professional research training, and 14% were actively pursuing a research career)	Citizen-led	X	X					X
Gražulevičienė et al[37]*<br>Gražulevičienė et al[38]*	Physical activity (Built environments, Green space), Health conditions (Hypertension)	X		580 citizen scientists (aged 18–75-years, gender n/a)	Co-created	X	X	X	X			
Gražulevičienė et al[39]	Physical activity (Built environments, Green space), Health conditions (Hypertension)		X	1062 citizen scientists (aged 18–75-years, 54% female, 29.6% living with chronic disease, 16% low income)	-	-	-	-	-			X
Hancock et al[40]	Diet and nutrition (Food environments); and Physical activity (Built environments)	X	X	5000 citizen scientists (characteristics n/a)	Co-created	X	X	X	X	X	X	X
Kapenekakis and Chorianopoulos[41]	Physical activity (Walkability)	X		13 citizen scientists (aged 19-25 years, 15% female)	Contributory				X			
Katapally et al[42]	Physical activity	X		301 citizen scientists (age n/a, 64% female)	Contributory		X			X		

* Articles reporting on same citizen science project are reported together and appear alphabetically by first published.

Katapally[43]	Physical activity and Mental health (Suicide prevention, substance use)	X	X	76 citizen scientists (aged 13-18 years, gender n/a, Indigenous)	Collaborative		X		X			X
Katapally and Chu[44]	Physical activity	X		89 citizen scientists (aged 18+ years, 52% female and 19% did not identify)	Collaborative	X	X		X			
Katapally et al[45]	Physical activity	X		71 citizen scientists (aged 18-48 years, 73% women, university students)	Collaborative	X	X					
Kim et al[46]	Diet and nutrition (Food environments); and Community health	X	X	12 citizen scientists (aged 13-18 years, members of the Karuk Tribe)	Co-created	X	X	X	X	X	X	X
Lehman et al[47]	Physical activity (Built environments)	X	X	12 citizen scientists (aged 20-60+, 67% female, previous volunteers)	Co-created		X					
Lehman et al[48]	Healthy work environments	X		541 citizen scientists (aged 21-67 years, living with multiple sclerosis)	Collaborative	X	X					
Lehnert et al[49]	Health and sustainability (Heat stress)	X		1525 citizen scientists (aged 11-81+ years, 50% women)	Contributory		X			X		
Lloret et al[50]	Health and sustainability (oceans and health)		X	n/a	Collaborative	-	-	-	-	-	X	
Modave et al[51]	Health conditions (mhealth for chronic condition self-management)	X		3 citizen scientists (age n/a, gender n/a, living with obesity and/or diabetes; paid volunteers from the UFCTSI[1] citizen science program).	Collaborative	X				X		
Moran et al[52]	Physical activity (Walkability)	X		59 citizen scientists (aged 50+ years, 76% female)	Collaborative		X					
Nelson et al[53]	Physical activity (Cycling safety)	X		n/a	Contributory		X			X		

[1] University of Florida Clinical and Translational Science Institute citizen science program

Odunitan-Wayas et al[54]	Physical activity (Built environments)	X	X	11 citizen scientists (aged 21–45 years, 73% female, 64% overweight/obese, low-income and mostly unemployed)	Collaborative		X	X				
Patel et al[55]	Community health	X	X	n/a	Co-created	X	X	X			X	X
Pedell et al[56]	Health conditions (mhealth for chronic condition self-management), Other (Social prescribing)	X	X	14 citizen scientists (including 8 in case study 1, aged 65+ years, 75% female, living with chronic conditions; and 6 in case study 2, aged 20-90 years)	Contributory	X	X			X	X	
Pollard, Roetman, and Ward[57]*<br>Csortan, Ward, and Roetman[58]*	Health and sustainability (Community gardens)	X		400 citizen scientists, including 34 citizen scientists who completed additional tasks (aged 18+ years, 71% female)	Contributory		X					
Richardson[59]	Drugs and alcohol (Alcohol misuse)	X	X	7 citizen scientists (including adults aged 50+ years, and youth aged 13-25 years, at- risk of harm from alcohol)	Co-created	X	X	X	X	X	X	X
Rodriguez et al[60]	Physical activity (School-based physical activity programmes)	X	X	32 citizen scientists; (including adults aged 46–49 years, 90% female (*n*=8); and youth aged 12-13 years, middle school students (*n*=26))	Collaborative		X	X	X	X	X	X
Roe et al[61]	Physical activity (Walkability) and Mental health	X	X	11 citizen scientists (aged 57-77 years, 46% female, low-income, 63% with a registered disability)	Contributory		X					
Rowbotham et al[62]	Healthy work environments (Workplace support for breastfeeding)	X	X	48 citizen scientists (aged 18-64 years, 94% women, 92% had children)	Contributory		X			X	X	

Rosas et al[63]	Physical activity (Built environments)	X	X	41 citizen scientists (32 adults aged 57 years on average and 9 adolescents aged 13 years on average; 71% female)	Collaborative		X	X		X		
Rubio et al[64]	Physical activity	X	X	48 citizen scientists (aged 34-70 years, 85% female, 50% with overweight/obesity)	Co-created		X	X	X	X	X	X
Rydenstam et al[65]*	Physical activity	X		24 citizen scientists (aged 16-19 years, 75% female; low socio-economic status)	Contributory		X					
Bälter et al[66]*												
Salloum et al[67]	Drugs and alcohol (Tobacco Prevention)	X		5 citizen scientists (aged 15-20 years, 60% female)	Collaborative	X				X		
Sarmiento et al[68]	Physical activity (Built environments)	X		28 citizen scientists (aged 18+ years)	Co-created		X	X	X	X		
Seguin et al[69]	Diet and nutrition (Food environments); and Physical activity (Built environments)	X		24 citizen scientists (aged 56-82 years, 58% female)	Contributory		X					
Sheats et al[70]	Physical activity (Walkability)	X		15 citizen scientists (including adolescents aged 11 -14 years (*n*=8), and older adults aged 63-80 years (*n*=7), 60% female, Latino)	Contributory		X					
Sheats et al[71]	Diet and nutrition (Food environments)	X	X	23 citizen scientists (aged 61-92 years, 70% female, low-income and food insecure)	Collaborative		X	X	X	X	X	X
Spitz et al[72]	Diet and nutrition (Artificial food additives)	X		12 citizen scientists (aged 18-45 years, 17% female)	Contributory	X						
Thoma et al[73]	Physical activity (Pedestrian safety)	X		25 citizen scientists (aged 16-60 years, 36% female)	Contributory	X						

Thomas et al[74]	Other (Alcohol advertising)		X	282 citizen scientists (aged 18-66+ years, 100% woman, 62% higher-level education)	-	-	-	-	-	X		
Townson et al[75]	Other (Community engagement in health research)	X	X	26 citizen scientists (aged 16+ years, gender n/a)	Collaborative	X			X			
Tuckett et al[76]	Physical activity (Built environments)	X	X	8 citizen scientists (aged 65+ years, 88% female, independently mobile)	Collaborative		X	X	X	X	X	
Tuckett et al[77]	Diet and nutrition (Food security initiative)	X	X	13 citizen scientists (aged 51–87 years, 92% female)	Collaborative		X	X	X	X	X	
Vandevijvere et al[78]	Diet and nutrition (Food environments)	X		45 citizen scientists (age n/a, 70% female)	Contributory				X	X		
Winter et al[79]	Physical activity (Built environments)	X	X	20 citizen scientists (including adolescents aged 12-13 years (*n*=10) and older adults aged 64-77 years (*n*=10), 80% female, low income, Latino)	Collaborative		X	X	X	X	X	
Winter et al[80]	Physical activity (Green space)	X		8 citizen scientists (aged <17-89 years, 88% female)	Contributory		X			X		
Zhao et al[81]	Health and sustainability (Heat stress)	X		68 citizen scientists (aged 19-76 years, 70% female, low-income)	Contributory		X			X		
Zieff et al[82]	Physical activity (Built environments)	X		50 citizen scientists (aged 18-89 years, 50% female, 5 were homeless)	Contributory		X					

Supplementary materials: Further references

1. Tricco AC, Lillie E, Zarin W, O'Brien KK, Colquhoun H, Levac D, et al. PRISMA extension for scoping reviews (PRISMA-ScR): checklist and explanation. Annals of internal medicine. 2018;169(7):467-73.
2. Abu Ali M, Alawadi K, Khanal A. The role of green infrastructure in enhancing microclimate conditions: A case study of a low-rise neighborhood in Abu Dhabi. Sustainability. 2021;13(8):4260.
3. Agostini G, SturtzSreetharan C, Wutich A, Williams D, Brewis A. Citizen sociolinguistics: A new method to understand fat talk. PloS one. 2019;14(5):e0217618.
4. Andersen TO, Dissing AS, Varga TV, Rod NH. The SmartSleep Experiment: Evaluation of changes in night-time smartphone behavior following a mass media citizen science campaign. PloS one. 2021;16(7):e0253783.
5. Armstrong B, Bridge G, Oakden L, Reynolds C, Wang C, Panzone LA, et al. Piloting Citizen Science Methods to Measure Perceptions of Carbon Footprint and Energy Content of Food. 2020;4(120).
6. Armstrong B, Reynolds C, Bridge G, Oakden L, Wang C, Panzone L, et al. How does Citizen Science compare to online survey panels? A comparison of food knowledge and perceptions between the Zooniverse, Prolific and Qualtrics UK Panels. Frontiers in Sustainable Food Systems. 2021:306.
7. Barrie H, Soebarto V, Lange J, Corry-Breen M, Walker L, editors. Using citizen science to explore neighbourhood influences on ageing well: Pilot project. Healthcare; 2019: Multidisciplinary Digital Publishing Institute.
8. Booth JN, Chesham RA, Brooks NE, Gorely T, Moran CN. A citizen science study of short physical activity breaks at school: improvements in cognition and wellbeing with self-paced activity. BMC Medicine. 2020;18(1):62.
9. Buman MP, Bertmann F, Hekler EB, Winter SJ, Sheats JL, King AC, et al. A qualitative study of shopper experiences at an urban farmers' market using the Stanford Healthy Neighborhood Discovery Tool. Public health nutrition. 2015;18(6):994-1000.
10. Buman MP, Winter SJ, Baker C, Hekler EB, Otten JJ, King AC. Neighborhood eating and activity advocacy teams (NEAAT): engaging older adults in policy activities to improve food and physical environments. Translational behavioral medicine. 2012;2(2):249-53.
11. Buman MP, Winter SJ, Sheats JL, Hekler EB, Otten JJ, Grieco LA, et al. The Stanford Healthy Neighborhood Discovery Tool: A Computerized Tool to Assess Active Living Environments. American Journal of Preventive Medicine. 2013;44(4):e41-e7.
12. Burcham ZM, Garneau NL, Comstock SS, Tucker RM, Knight R, Metcalf JL. Patterns of oral microbiota diversity in adults and children: a crowdsourced population study. Scientific reports. 2020;10(1):1-15.
13. Cambria VE, Campagnaro T, Trentanovi G, Testolin R, Attorre F, Sitzia T. Citizen Science Data to Measure Human Use of Green Areas and Forests in European Cities. Forests. 2021;12(6):779.
14. Cervinka R, Schwab M, Haluza DJIJoER, Health P. Investigating the Qualities of a Recreational Forest: Findings from the Cross-Sectional Hallerwald Case Study. 2020;17(5):1676.
15. Chesser SA, Porter MM, Barclay R, King AC, Menec VH, Ripat J, et al. Exploring University Age-Friendliness Using Collaborative Citizen Science. The Gerontologist. 2020;60(8):1527-37.
16. Chrisinger BW, King AC. Stress experiences in neighborhood and social environments (SENSE): a pilot study to integrate the quantified self with citizen science to improve the built environment and health. International journal of health geographics. 2018;17(1):17.

17. Chrisinger BW, Ramos A, Shaykis F, Martinez T, Banchoff AW, Winter SJ, et al. Leveraging citizen science for healthier food environments: a pilot study to evaluate corner stores in Camden, New Jersey. Frontiers in public health. 2018;6:89.
18. Clotworthy A, Dissing AS, Nguyen T-L, Jensen AK, Andersen TO, Bilsteen JF, et al. 'Standing together–at a distance': Documenting changes in mental-health indicators in Denmark during the COVID-19 pandemic. Scandinavian Journal of Public Health. 2020:1403494820956445.
19. Collins S, Brueton R, Graham TG, Organ S, Strother A, West SE, et al. Parenting Science Gang: radical co-creation of research projects led by parents of young children. Research Involvement and Engagement. 2020;6(1):1-15.
20. De Cocker K, Chastin SF, De Bourdeaudhuij I, Imbo I, Stragier J, Cardon G. Citizen science to communicate about public health messages: the reach of a playful online survey on sitting time and physical activity. Health communication. 2019;34(7):720-5.
21. Den Broeder L, Lemmens L, Uysal S, Kauw K, Weekenborg J, Schönenberger M, et al. Public health citizen science; perceived impacts on citizen scientists: A case study in a low-income neighbourhood in the Netherlands. Citizen Science: Theory and Practice. 2017;2(1).
22. Díaz JL, Codern-Bové N, Zomeño M-D, Lassale C, Schröder H, Grau M. Quantitative and qualitative evaluation of the COMPASS mobile app: A citizen science project. Informatics for Health and Social Care. 2021;46(4):412-24.
23. Díez J, Gullón P, Sandín Vázquez M, Álvarez B, Martín M, Urtasun M, et al. A community-driven approach to generate urban policy recommendations for obesity prevention. International journal of environmental research and public health. 2018;15(4):635.
24. Dilley JR, Moore JB, Summers P, Price AA, Burczyk M, Byrd L, et al. A Citizen Science Approach to Determine Physical Activity Patterns and Demographics of Greenway Users in Winston-Salem, North Carolina. International journal of environmental research and public health. 2019;16(17):3150.
25. Dissing AS, Andersen TO, Nørup LN, Clark A, Nejsum M, Rod NH. Daytime and nighttime smartphone use: A study of associations between multidimensional smartphone behaviours and sleep among 24,856 Danish adults. Journal of Sleep Research. 2021;30(6):e13356.
26. Dobson MC, Reynolds C, Warren PH, Edmondson JL. "My little piece of the planet": the multiplicity of well-being benefits from allotment gardening. British Food Journal. 2020.
27. Dolejšová M, Kera D, editors. Soylent Diet Self-Experimentation: Design Challenges in Extreme Citizen Science Projects. Proceedings of the 2017 ACM Conference on Computer Supported Cooperative Work and Social Computing; 2017: ACM.
28. Eames M, Egmose J. Community foresight for urban sustainability: Insights from the Citizens Science for Sustainability (SuScit) project. Technological Forecasting and Social Change. 2011;78(5):769-84.
29. Edmondson JL, Childs DZ, Dobson MC, Gaston KJ, Warren PH, Leake JR. Feeding a city–Leicester as a case study of the importance of allotments for horticultural production in the UK. Science of the Total Environment. 2020;705:135930.
30. Fancourt D, Baxter L, Lorencatto F. Barriers and enablers to engagement in participatory arts activities amongst individuals with depression and anxiety: quantitative analyses using a behaviour change framework. BMC public health. 2020;20(1):1-12.
31. Fell T, Rydenstam T, Buli BG, King AC, Bälter K. Citizen Science in Sweden's Stigmatized Neighborhoods. Sustainability. 2021;13(18):10205.
32. Folkerth M, Adcock K, Singler M, Bishop E. Citizen Science: A New Approach to Smoke-Free Policy Advocacy. Health Promotion Practice. 2020;21(1_suppl):82S-8S.
33. Frei A, Dalla Lana K, Radtke T, Stone E, Knöpfli N, Puhan MA. A novel approach to increase physical activity in older adults in the community using citizen science: a mixed-methods study. International journal of public health. 2019:1-10.

34. González SA, Rubio MA, Triana CA, King AC, Banchoff AW, Sarmiento OL. Building healthy schools through technology-enabled citizen science: The case of the o ur voice participatory action model in schools from Bogotá, Colombia. Global Public Health. 2021:1-17.
35. Graham S, Zha CC, King AC, Banchoff AW, Sarnquist C, Dauber M, et al. A novel model for generating creative, community-responsive interventions to reduce gender-based violence on college campuses. International journal of Environmental Research and Public Health. 2021;18(15):7933.
36. Grant AD, Wolf GI, Nebeker C. Approaches to governance of participant-led research: a qualitative case study. BMJ open. 2019;9(4):e025633.
37. Gražulevičienė R, Andrušaitytė S, Dėdelė A, Gražulevičius T, Valius L, Kapustinskienė V, et al. Environmental quality perceptions and health: A cross-sectional study of citizens of Kaunas, Lithuania. International journal of Environmental Research and Public Health. 2020;17(12):4420.
38. Grazuleviciene R, Andrusaityte S, Gražulevičius T, Dėdelė A. Neighborhood Social and Built Environment and Disparities in the Risk of Hypertension: A Cross-Sectional Study. International journal of Environmental Research and Public Health. 2020;17(20):7696.
39. Grazuleviciene R, Andrusaityte S, Rapalavicius A. Measuring the Outcomes of a Participatory Research Study: Findings from an Environmental Epidemiological Study in Kaunas City. Sustainability. 2021;13(16):9368.
40. Hancock C, Clarke S, Stevens D. Supporting individuals' healthy eating requires genuine engagement with communities. Nutrition Bulletin. 2019;44(1):92-9.
41. Kapenekakis I, Chorianopoulos K. Citizen science for pedestrian cartography: collection and moderation of walkable routes in cities through mobile gamification. Human-centric Computing and Information Sciences. 2017;7(1):10.
42. Katapally TR, Bhawra J, Leatherdale ST, Ferguson L, Longo J, Rainham D, et al. The SMART study, a mobile health and citizen science methodological platform for active living surveillance, integrated knowledge translation, and policy interventions: Longitudinal Study. JMIR public health and surveillance. 2018;4(1):e31.
43. Katapally TR. Smart Indigenous Youth: The Smart Platform Policy Solution for Systems Integration to Address Indigenous Youth Mental Health. 2020;3(2):e21155.
44. Katapally TR, Chu LM. Digital epidemiological and citizen science methodology to capture prospective physical activity in free-living conditions: a SMART Platform study. BMJ open. 2020;10(6):e036787.
45. Katapally TR, Hammami N, Chu LM. A randomized community trial to advance digital epidemiological and mHealth citizen scientist compliance: A smart platform study. Plos one. 2021;16(11):e0259486.
46. Kim KK, Ngo V, Gilkison G, Hillman L, Sowerwine J, Leaders KY. Native American Youth Citizen Scientists Uncovering Community Health and Food Security Priorities. Health promotion practice. 2019:1524839919852098.
47. Lehman E, Jepson R, McAteer J, Archibald D. What motivates volunteers to engage in health-related citizen science initiatives? A case study of our outdoors. International Journal of Environmental research and Public Health. 2020;17(19):6950.
48. Lehmann AI, Rodgers S, Kamm CP, Mettler M, Steinemann N, Ajdacic-Gross V, et al. Factors associated with employment and expected work retention among persons with multiple sclerosis: findings of a cross-sectional citizen science study. Journal of neurology. 2020;267(10):3069-82.
49. Lehnert M, Geletič J, Kopp J, Brabec M, Jurek M, Pánek J. Comparison between mental mapping and land surface temperature in two Czech cities: A new perspective on indication of locations prone to heat stress. Building Environment. 2021;203:108090.

50. Lloret J, Abós-Herràndiz R, Alemany S, Allué R, Bartra J, Basagaña M, et al. The roses ocean and human health chair: A new way to engage the public in oceans and human health challenges. International journal of environmental research. 2020;17(14):5078.
51. Modave F, Bian J, Rosenberg E, Mendoza T, Liang Z, Bhosale R, et al. DiaFit: the development of a smart app for patients with type 2 diabetes and obesity. JMIR diabetes. 2016;1(2):e5.
52. Moran MR, Werner P, Doron I, HaGani N, Benvenisti Y, King AC, et al. Exploring the Objective and Perceived Environmental Attributes of Older Adults' Neighborhood Walking Routes: A Mixed Methods Analysis. Journal of aging and physical activity. 2017;25(3):420-31.
53. Nelson TA, Denouden T, Jestico B, Laberee K, Winters M. BikeMaps.org: A Global Tool for Collision and Near Miss Mapping. Frontiers in Public Health. 2015;3(53).
54. Odunitan-Wayas FA, Hamann N, Sinyanya NA, King AC, Banchoff A, Winter SJ, et al. A citizen science approach to determine perceived barriers and promoters of physical activity in a low-income South African community. Global Public Health. 2020:1-14.
55. Patel DI, Winkler P, Botello J, Villarreal J, Puga F. The citizen scientist: Community-academic partnerships through Translational Advisory Boards. Patient Education and Counseling. 2016;99(12):2087-90.
56. Pedell S, Borda A, Keirnan A, Aimers N. Combining the digital, social and physical layer to create age-friendly cities and communities. International Journal of Environmental Research and Public Health. 2021;18(1):325.
57. Pollard G, Roetman P, Ward J. The case for citizen science in urban agriculture research. Future of Food: Journal on Food, Agriculture and Society. 2017;5(3):9-20.
58. Csortan G, Ward J, Roetman P. Productivity, resource efficiency and financial savings: An investigation of the current capabilities and potential of South Australian home food gardens. PloS one. 2020;15(4):e0230232.
59. Richardson L. Engaging the Public in Policy Research: Are Community Researchers the Answer? Politics and Governance. 2014;2(1):32-44.
60. Rodriguez NM, Arce A, Kawaguchi A, Hua J, Broderick B, Winter SJ, et al. Enhancing safe routes to school programs through community-engaged citizen science: two pilot investigations in lower density areas of Santa Clara County, California, USA. BMC public health. 2019;19(1):256.
61. Roe J, Mondschein A, Neale C, Barnes L, Boukhechba M, Lopez S. The urban built environment, walking and mental health outcomes among older adults: a pilot study. Frontiers in Public Health. 2020;8:528.
62. Rowbotham S, Marks L, Tawia S, Woolley E, Rooney J, Kiggins E, et al. Using citizen science to engage the public in monitoring workplace breastfeeding support in Australia. Health Promotion Journal of Australia. 2021.
63. Rosas LG, Salvo D, Winter SJ, Cortes D, Rivera J, Rodriguez NM, et al. Harnessing technology and citizen science to support neighborhoods that promote active living in Mexico. Journal of Urban Health. 2016;93(6):953-73.
64. Rubio MA, Triana C, King AC, Rosas LG, Banchoff AW, Rubiano O, et al. Engaging citizen scientists to build healthy park environments in Colombia. Health Promotion International. 2020.
65. Rydenstam T, Fell T, Buli BG, King AC, Bälter K. Using citizen science to understand the prerequisites for physical activity among adolescents in low socioeconomic status neighborhoods-The NESLA study. Health & Place. 2020;65:102387.
66. Bälter K, Rydenstam T, Fell T, King AC, Buli BG. Data from an Our Voice citizen science initiative in neighborhoods with low socioeconomic status in Sweden: A proof of concept for collecting complex data. Data in Brief. 2020;33:106394.

67. Salloum RG, Theis RP, Pbert L, Gurka MJ, Porter M, Lee D, et al. Stakeholder Engagement in Developing an Electronic Clinical Support Tool for Tobacco Prevention in Adolescent Primary Care. Children. 2018;5(12):170.
68. Sarmiento OL, Higuera-Mendieta D, Wilches-Mogollon MA, Guzman LA, Rodriguez DA, Morales R, et al. Urban Transformations and Health: Methods for TrUST—a Natural Experiment Evaluating the Impacts of a Mass Transit Cable Car in Bogotá, Colombia. Frontiers in public health. 2020;8:64.
69. Seguin RA, Morgan EH, Connor LM, Garner JA, King AC, Sheats JL, et al. Peer Reviewed: Rural Food and Physical Activity Assessment Using an Electronic Tablet-Based Application, New York, 2013–2014. Preventing chronic disease. 2015;12.
70. Sheats J, Winter S, Padilla-Romero P, Goldman-Rosas L, Grieco L, King A, editors. Comparison of passive versus active photo capture of built environment features by technology naïve Latinos using the SenseCam and Stanford healthy neighborhood discovery tool2013 2013.
71. Sheats J, Winter S, Padilla-Romero P, King A. FEAST: empowering community residents to use technology to assess and advocate for healthy food environments. Journal of Urban Health. 2017;94(2):180-9.
72. Spitz R, Junior CP, Queiroz F, Leite LC, Dam P, Rezende AC. Gamification, citizen science, and civic technologies: In search of the common good. Strategic Design Research Journal. 2018;11(3):263-73.
73. Thoma M, Theodosiou Z, Partaourides H, Tylliros C, Antoniades D, Lanitis A, editors. A Smartphone Application Designed to Detect Obstacles for Pedestrians' Safety. International Summit Smart City 360°; 2020: Springer.
74. Thomas JA, Trigg J, Morris J, Miller E, Ward PR. Exploring the potential of citizen science for public health through an alcohol advertising case study. Health promotion international. 2021.
75. Townson J, Davies J, Hurt L, Ashfield-Watt P, Paranjothy S. Developing and evaluating a model of public involvement and engagement embedded in a national longitudinal study: HealthWise Wales. International Journal of Population Data Science. 2020;5(3).
76. Tuckett A, Freeman A, Hetherington S, Gardiner P, King A. Older Adults Using Our Voice Citizen Science to Create Change in Their Neighborhood Environment. International journal of environmental research and public health. 2018;15(12):2685.
77. Tuckett AG, Rowbotham S, Hetherington S, Goddard J, King AC. Using citizen science to empower older adults to improve a food security initiative in Australia. Health Promotion International. 2022;37(1):daab060.
78. Vandevijvere S, Williams R, Tawfiq E, Swinburn B. A food environments feedback system (FoodBack) for empowering citizens and change agents to create healthier community food places. Health promotion international. 2017;34(2):277-90.
79. Winter SJ, Rosas LG, Romero PP, Sheats JL, Buman MP, Baker C, et al. Using citizen scientists to gather, analyze, and disseminate information about neighborhood features that affect active living. Journal of immigrant and minority health. 2016;18(5):1126-38.
80. Winter SJ, Sheats JL, Salvo D, Banda JA, Quinn J, Rivera BR, et al. A Mixed Method Study to Inform the Implementation and Expansion of Pop-Up Parks for Economic, Behavioral, and Social Benefits. Journal of Urban Health. 2020;97(4):529-42.
81. Zhao Q, Li Z, Shah D, Fischer H, Solís P, Wentz E. Understanding the interaction between human activities and physical health under extreme heat environment in Phoenix, Arizona. Health & Place. 2021:102691.
82. Zieff SG, Musselman EA, Sarmiento OL, González SA, Aguilar-Farias N, Winter SJ, et al. Talking the walk: perceptions of neighborhood characteristics from users of Open Streets Programs in Latin America and the USA. Journal of urban health. 2018;95(6):899-912.
